# Panel data analysis of profitability and employment growth of medium and large size industries in Ethiopia

**DOI:** 10.1016/j.heliyon.2022.e10859

**Published:** 2022-10-04

**Authors:** Aragaw Eshetie Aguade, Dasash Ayanaw, Endeshaw Assefa Derso

**Affiliations:** a Department of Statistics, Under Natural and Computational Science College, University of Gondar, Gondar, Ethiopia; b Department of Statistics , Under Natural and Computational Science College, Aksum University, Ethiopia

**Keywords:** Manufacturing industries, Firm profitability, Employment growth, Panel data, Random estimation technique

## Abstract

Manufacturing industries are an asset or value added part of an economy, intimately linked with industrial and engineering design, and engaged in producing significant economic productions that can assist the national growth and development. The aim of the study was to estimate the profitability and employment growth of medium and large size industries in Ethiopia. The variables were chosen based on theoretical and experimental literature findings. In this study, an explanatory study design was implemented in carrying out this research with the prearrangement of secondary data collected from the panel data set of medium and large size manufacturing industries conducted annually by the central statistics agency over the period 2002–2011 E.C. The study employed panel data estimation methods to analyze the influence of medium and large size manufacturing industries on profitability and employment growth. Three panel data models are used: pooled ordinary least squares, fixed effect, and random effect estimation method. The Hausman test revealed the random model was the best fit for both profitability and employment growth. The diagnosticstest: normality, multicollinearity, heteroscedasticity, and autocorrelation tests were conducted on the data. The results obtained indicate that employment growth and profitability in Ethiopia are generally driven by medium and large-size manufacturing industries. The random model result shows that ownership, firm-size, advertising intensity and import intensity have a positive influence and substantial effect on profitability. But, the government has significant effect and a negative influence on profitability. The random model shows that government, advertising intensity and firm-size have significant effect and positive influence on employment growth. Nevertheless, import has significant effect and a negative influence on employment growth. Eventually, the employment growth and profitability have a positive influence and insignificant effect and the authors suggested further research in the areas by taking into account additional variables and newly emerging industries.

## 1 Introduction

### 1.1 Preamble

Manufacturing industries are a wealth making of the economic sector, thoroughly linked with industrial plan and engineering, and engaged in stipulating significant profitable productions which can assist the Economic development of the country. The manufacturing evolution may involve chemical or mechanical conversion of materials or substances into new outputs. But, manufacturing is a procedure uses manual labor or machinery to create things from raw materials, and it is usually done in a methodical manner with a partition of labor. In other way, manufacturing firm is the assembly or production of goods into ended products on an impartially large size manufacturing haven’t included cottage industries. The manufacturing denotes to those firms which are presume in the processing and manufacturing of items, value addition or the making of new commodities to standard supplies through chemical or mechanical transformation (Bataille et al., 2018).

Global manufacturing industries have, for the past two decades, been considered by a significantly great pace of progress in the least developed countries compared to industrialized countries, which is generally explained by external and domestic factors. The low production base, an increasing surplus labour force, an abundance of locally produced raw materials, domestic investment, and pro-growth government policies helped as major domestic factors in increasing manufacturing in developing countries. The external determinant factors comprised foreign direct investment with a strong gain of outsourcing production activities by companies focused in industrialized countries and favourable situations in international trade. Whereas, manufacturing growth slow down in industrialized countries for three reasons. First, economic growth has slowed significantly as a consequence of market saturation. Second, manufacturing has shifted to emerging countries; third, as income improved, the service sector increased quickly and squeezing manufacturing’s share, took a larger proportion of the economy (Stuckler et al., 2012).

The manufacturing industry in Sub-Saharan part of Africa has been shrinking or stagnant for the previous 30 years (Bigsten and Gebreeyesus, 2009). From the perspective of poverty alleviation, jobs creations, supporting non-industrial divisions, and the sectors' situations are still troublesome. As (Bigsten and Gebreeyesus, 2009) and others have argued, Africa’s industrial growth has been hampered by a slew of difficulties of high transaction and transportation costs due to insufficient evidence and deal execution, in addition very dangerous political and economic conditions. Furthermore, the progress of grassroots organizations, social assets, and the establishment of public services are considered to be inadequate in Africa (Musavengane and Simatele, 2017). In recent times, African countries have confirmed a renewed promise to industrial development as a part of broader agenda to improve productive capacity for continuous and high economic growth, produce employment opportunities and significantly reduce poverty. However, it still contained for a depressed share of worldwide manufacturing (Hitimana et al., 2010).

Firms conduct the most profitable initiatives initially, according to old microeconomic theories, before continuing their expansion with less profitable ones. As a company increases in size, it suit more difficult for its managers to increase its growth and profitability (Steffens et al., 2009). When firm grows in size, more organizational and management complexity emerges, diverting managers' attention away from controlling operational expenses and resulting in a decline in profit rate. As a result, profitability cannot be achieved through just growth. Evolutionary theories on business growth, led by the study by (Cornell and Kalt, 2005), argue that profitability increase the expansion and development of the company. They contend that more suited firms survive and flourish, whereas less feasible firms deteriorate till they shut for good. In the same manner, although with different opinions, the resource centered view (RCV) theory upholds that firms showing considerable profitability amounts are more probable to grow.

### 1.2 Brief on Ethiopia’s manufacturing sector

When we look at the history of the Ethiopia manufacturing firm, we can see that it dates back in 1920s, it was an easy and a simple technology which produces agriculture centered goods and products. However, the official institutionalization exertion or the foundation of the manufacturing business industry sector in Ethiopia was started in the early 1960’s and late 1950’s when the imperial regime established a new strategy to beach up the nation economy by inviting foreign direct investments, mostly in the manufacturing segment. Tax exemptions, tax-free exports and imports, transfer of foreign exchange, monetary provision from the nation investment enterprise and tax exemptions on dividends were part of the new policy. Furthermore, the government enforced protective actions for industries by imposing high tariffs on commodities that could reduce the company market slice of locally manufactured products. The government’s asset was nearly in all industrial clusters, like the beverage and food, pulp and paper, bottle and glass, cement industries, tires and textile industries etc. However, the manufacturing industry remains in its infant phase, dominantly concentrating on semi-processing section of the sectors. Several interconnected factors have combined to obstruct the development of a more robust manufacturing concentrated base economy in Ethiopia (Yayeh, 2018). Still, the Ethiopian governments have tried to develop the means of altering those problems by using the nation’s different justifiable benefits in the sector, including the cheap and simply trainable labor force, expansion of raw material and development of infrastructures and utility commodities. The government has created a favorable legal basis for the development of the manufacturing industry, with the goal of driving the nation’s economic development and growth through horizontal and vertical relations or links based on the country’s abundant agricultural and mineral resources (Fikru, 2014).

In general, the Ethiopian large and medium size manufacturing industry is described by its deprived performance with regard to its jobs creation, role to the general national income, kind of goods and products produced, and so on. According to (Broberg et al., 2020) regardless of the entire number of industries has improved by a certain quantity in this time, labor employment has presented only an irrelevant conversion. The most likely explanation offered for the weak employment growth is that industries are not functioning at their full capabilities, which is extremely related to the performance of profitability of the manufacturing firms. Presently, Ethiopian manufacturing firms are producing 50% of their capabilities that leads to deprived resource usage and lower levels of employment growth and profitability (Endale, 2011). In this regard, the sector has a difficult with viable growth and profitability since Ethiopians' aggregate economic wellbeing and individual livelihoods depend mainly on the subsistence agrarian economy and the external budget that occupy greater than 80% of the economically matured energetic population, while the manufacturing area in particular and the industry sector in general hold very few jobs (Diriba, 2018).

#### 1.2.1 Manufacturing industries in Ethiopian situation

According to Stephen and Wasiu (2013), when defining medium and large size enterprises and industries, references are made to qualitative and numerical measures including the number of people employed in the enterprises or industries, investment outlay, annual sales turnover and a combination of these measures. Taking in to consideration, the classification and definition of industries in the context of our country are discussed below. This enterprise or industrial company classification is based on Ethiopia’s new medium and large enterprise development strategy (LMEDSE), 2011. This classification of industrial company size is mainly based on work force (man power) and 11 capitals, which include machinery costs and 19 working capital but exclude land and buildings costs of Industry.

The following is the arrangement and definition: Micro Enterprises are defined as businesses with less than five employees, including the owner, and total assets of fewer than 100,000 ETB in the industrial sector. Small size manufacturing is an industrial company that employs 6–30 people and has assets ranging from 100,001 to 1,500,000 ETB. Medium scale industries an industrial company with 31–200 employees with a total asset of 1,500,001 to 30,000,000 ETB, and Large scale industries an industrial company with greater than 200 employees with a total asset of more than 30,000,000 ETB.

Ethiopia’s government has devised and implemented long-term, medium-term, and short-term plans to alleviate poverty and ensure rapid and sustainable economic development in a variety of sectors. The government believes that industrial growth is a critical tool for ensuring rapid and sustainable economic development. As a result, create an Industrial Strategy and a five-year Development and Transformation Plan to accelerate the transition from agriculture to an industry-led economy. Leather industry, textile and garment, agro-processing, chemical, pharmaceutical, metal industry, meat and milk industry were the prioritized subsectors based on GTP of Manufacturing industry sector. So far, the above subsectors have provided various supports and coordinated efforts to achieve the GTP goals. In its Development and Transformation Plan (2010/11–2014/15), the Ethiopian government emphasized the importance of ensuring rapid and sustainable development of the industrial sector.

The Ethiopian manufacturing industry sector is controlled by beverages, food products and non-metallic mineral manufacturing sub-sectors. The manufacturing of beverages and food products industrial group. Industries such as metal processing, chemical, electronics, and other engineering manufacturing industries, which build technical competences and dynamism, haven’t yet been developed (CSA, 2018). Most manufacturing exports are centered on farming, dress, counting drinks, shoes, and semi-processed skins. Moreover, most capital and manufactured consumer goods are imported into Ethiopia, which is additionally intensely dependent on the importation of fuel. The government is committed to making a favorable environment for inviting foreign direct investment and Promoting domestic investment. An assortment of outside companies from, India, China, Japan and Turkey are directly competing within the nation to use this opportunity. The special duty free exchange get to given by Ethiopia to European Union and the United States of America markets to affords strategic opportunities (Hailu and Tanaka, 2015).

Ethiopia has plenteous assets that can give profitable inputs for light fabricating, to be specific, cattle, which can be used as an input for making leather products; forests, timberlands, which can be used as an input for the furniture industry; cotton, which could be used as an input for the clothing industry; and agricultural land and lakes are utilized to supply inputs for agro-processing businesses (Dinh et al., 2012). Besides, Ethiopia has ample low-cost labor, which gives it a relative advantage in labor-intensive and less-skilled divisions (Dinh et al., 2012).

### 1.3 Identified study gaps in the review of literature

As per the researcher’s possible effort to review the studies conducted on factors affecting profitability and employment growth in the medium and large-scale manufacturing industry, the problems haven’t been properly investigated. Whereas taking into account or consideration the lack of sufficient empirical evidence, the medium and large -scale manufacturing industries sector in Ethiopia do not have adequate financial resources, and the cause of taxation policy sometimes discourages the owners. In this case, some manufacturing industries stop work (Yilma et al., 2011).

The previous researcher used two general major factors, such as: global and national forces and industry factors. Those factors only affected the profitability of the small, medium, and large manufacturing industries sector in Ethiopia for the periods 1997–2006. However, this study aims to top up the gap by including two other major factors, such as finance availability and taxation policy, that have not been addressed by other researchers on both profitability and employment growth in Ethiopia. In addition, the previous researchers used the univariate panel data model, which means that the firm’s profitability was used as the outcome variable. However, this study used as a multivariate panel data model or the firms' employment growth and profitability were used as the outcome variables (Haftu, 2009).

First, if adequate financial assets are not available to apply technological changes and provide proper training to employees, an industrial unit’s ambitious objectives to expand employment growth and profitability would remain mere dreams. The greater the degree of modernization to be implemented, the bigger the capital requirement. Capital will also be necessary for research and development, advertising campaigns, improved working conditions for employees, and maintenance of plant and machinery, among other thing (Degefa, 2017).

Second, the Ethiopian taxation policy is an important source of funds for the provision of public services and the growth of the economy. The difficulties challenged are in the negative connection between taxes and businesses' ability to sustain them and to expand. LMSMI is faced with the difficult of multiple taxations, high tax rates, complex tax regulations, and a lack of proper information about tax related (Chanie, 2018).

Ethiopia is also well-known its diversifying range of mineral resources. Mineral exploitation and exploration are still in their infancy; hence their use has yet to be realized. This frustrated the enlargement of manufacturing businesses depend on mineral resources which resulted in lower profitability for those who are previously established. Likewise, the survey result on the manufacturing subdivision made by the commission for national plan preparation tells there is a significant difference between the cos of introduced raw materials and the cost of all manufacturing industrial raw materials used by industrial manufacturing enterprises with positive impact on the development of manufacturing industries (Yayeh, 2018). Besides, import raw material processes for foreign attained inputs or raw materials are very extended and bureaucratic. All of this added up to substantial and unfavorable associations and substantial influences on the progression of industries (Asare et al., 2017).

According to Abraham (2018) to Kaldor’s law, manufacturing is indeed the engine of the nation’s development, based on the abovementioned scholar, manufacturing is focus to increasing returns, together with dynamic and static while small and land-based industries are subject to diminishing rates of return. Furthermore, the author argued that the manufacturing industry aims to expand by recruiting labor from many other areas of the economy whose profits are declining. In this scenario, production increases gradually since the average labor production exceeds the marginal revenue. When such a result, as when the production of the industry develops, so does production growth in the economy, which is a fundamental determinant of GDP and people’s quality of life (Pacheco-López and Thirlwall, 2013).

According to firm theory, size is not the only factor influencing firm growth. Labor quality, innovation, productivity, financial performance, capital intensity, book value of total assets, legal form, ownership of firms, age, and other industry specific factors all influence firm growth. From a policy standpoint, this suggests that larger industries should have been targeted in order to increase employment (Hitt et al., 2000).

Larger firms play a significant role because of use of economies of scale, their higher productivity, their own technological capability, their propensity to generate high-paying secure jobs, and their own competitiveness in the export industry. Nevertheless, the numerical supremacy of small firms currently one of the main features of Ethiopia’s manufacturing sector (Lawrence 2005; Dinh et al., 2012).

According to a modern theory of firms, the key objective of a manufacturing industry is to optimize profits or minimize costs, particularly in a privately held company. This indicates that profit as a motivator for firm growth and as the objective of the firm’s survival is determined by external factors beyond the firm’s control (Sangosanya, 2011).

According to the new structural economics, industrial structure is inherent to endowment structure, and the differences between developing and developed countries are caused by investment fund base, and yet a developing country are becoming developed by shifting its industrial structure. With structural transformation comes infrastructure modernization to accommodate the newly established structure, which leads to industrial growth, rising incomes, and, ultimately, reducing poverty (Lin, 2010).

### 1.4 Objectives of the study

The main objective of the study was to evaluate the profitability and employment growth of the medium and large -scale manufacturing industry in Ethiopia.

To address the above main objective, this study has the following objectives.

• To identify the factors affecting the profitability and employment growth of large and medium-scale manufacturing industries from the period 2002–2011.E.C.
• To evaluate the relationship between profitability and employment growth of LMSMI.
• To identify appropriate estimator of the panel data regression model.

### 1.5 Significance of the study

This inquiry was designed to investigate the significant effects of time-varying and time invariant factors on profitability and employment growth of manufacturing industries in Ethiopia. The following are identified as contributions of the study:

• It provides evidence to any responsible organizations to design strategies, set policies, and look for resolutions on how to increase the profitability and employment growth of those firms/industries.
• It demonstrates that most researchers, particularly econometricians and statisticians, lack a fundamental understanding of panel data methods of estimation in general. Therefore, this study used to understood the different categories of panel data techniques.
• Based on empirical data, the research has identified the factors that influence profitability and employment growth of firms/industries. As a result, it supports the key results by offering additional empirical evidence.
• Based on the implication of the research findings, the research also recommended areas for future research.

## 2 Methodology

### 2.1 Study area

This inquiry was conducted in Ethiopia**.** Its location bounces it strategic supremacy as a bounding off point of the east Africa and near to the Middle East. Ethiopia is landlocked, neighboring Somalia, Eritrea, South Sudan, Sudan, Kenya, and has been using Djibouti’s key port for the past two decades. But, now Ethiopia has reached an agreement with Eritrea, Ethiopia will be able to resume using the Eritrean ports of Massawa and Assab for international trade with more than 112 million people (2019). Ethiopia is the most populous country in Africa after Nigeria, and the fastest increasing economy in the continent (Kidane, 2020).

### 2.2 Research design

This study used econometric methods focused on the set of panel numerical figures or data gathered from large and medium-scale manufacturing industries for profitability and employment growth. This research adopted an explanatory study design with the arrangement of secondary data collection followed by quantitative methods. The survey was implemented in a deductive manner, based on existing theories and research testing, the connection between the explanatory variables of profitability and employment growth. So the data collection procedure has been prepared in a format that is suitable for econometric analysis of the panel data analysis.

### 2.3 Data source

This study used a secondary panel data set on LMSMI conducted annually by the Central Statistics Agency (CSA) over the study period 2002–2011 E.C. This survey covered both private and public manufacturing industries in all regions of the country.

### 2.4 Sampling techniques

Based on the ISIC, the manufacturing industry sector has twenty-four industrial groups, but for the study purpose, the study used a sample of fifteen samples out of twenty-four manufacturers in Ethiopia because the study was using LMSMI. The LMSMI includes only fifteen industrial groups, which are listed in below. Therefore, the study covered an age of 10 years from 2002 to 2011 and the sample was LMSMI of Ethiopia. The study employed a purposive random sampling method to select the sample of the population and achieve the predefined objective of this study. The matrix for the panel data layout is 15(industry)∗10(years) that includes 150 observations.

### 2.5 Inclusion and exclusion criteria

This study considered the medium and largest scale manufacturing industry from the period of 2002 to 2011. This study has been comprised in the part of the medium and large size manufacturing industry that think about the size or number of employees is ten or more than ten. However, the study excludes that the number of industry employees is less than ten (10).

### 2.6 Study variables

In this study, two cohort variables were used:

#### 2.6.1 Response variable

Profitability or ROA (return on assets) and employment growth (EMG)

#### 2.6.2 Explanatory variables

• ***Time-varying***: import intensity (IMP), firm size (F-size), advertising intensity (ADVI), capital intensity (KPI)
• ***Time-Invariant*:** Ownership structure –dummy (OWN) and Government –dummy (GOV).

### 2.7 Panel data analysis

The following regression equation is the basic model for the panel data model:

(2.1) $$Y_{it}=\alpha +\beta x_{it}+\gamma z_{i}+\upsilon_{i}+\varepsilon_{it},$$

where, for unit i at time t, $y_{it}$ , $X_{it}$ and $Z_{i}$ are the values of the response variable y and the time varying independent variables x and time invariant independent variable z, respectively; β and γ are the regression coefficients; α is a constant intercept; variable varying only with the unit i, with variance; $\delta_{u}^{2}$ and $\varepsilon_{it}$ is a random term in an equation (that varies with unit and time).

**Assumption**(1) αis the constant, which is homogenous among different observations. (2) The influence of X on Y is different cross-sectional units and through time and Z on Y is constant time but not the same cross-sectional elements. (3) The random terms ($\varepsilon_{it}$) is uncorrelated and homoscedastic between cross-sectional elements and over time but the $cov\left(X,u_{i}\right)\;$ are not the same as zero.

Time-invariant explanatory variables is homogeneous across individuals since in varies cases, there are no unobservable individual-specific effects. The model parameters slope (β) and intercept (α), can be directly computed using pooled ordinary least squares. The fixed effects model includes the estimation of the model parameters, coefficient explanatory variables (β) and individual α_i_ for each of the N groups in the panel. However, the intercept is the same across industries, this estimation method is called the LSDV.

The random model is different from the Pooled (constant effect) ordinary least square (OLS) and the fixed effect models, especially since this model does not use the principle of OL squares but rather uses the principle feasible general least squares. However, this does not result in biased OLS coefficient estimations, but it does result in inefficient parameters and inaccurate standard inference tools (Saha et al., 2021).

Until now we have used three different types of estimation methods for panel data: random effects (RE), fixed effects (FE), & (Pooled) constant effects. The error or random terms in these methods that satisfy the assumptions of OL square if the separate model is correct. The LM test is aimed to determine whether a random effect or a simple OLS regression should be performed. The chow test statistic was used to compare the pooled model to the alternative hypothesis of the fixed model.

The Hausman test was used to compares the random versus fixed effects that the specific effects are independent with the regression parameters in the model of OLS (Amini et al., 2012). Tests for the (CLRM) assumptions to maintain the robustness and the data validity of the regressed results of the research, the basic CLRM assumptions have been verified to identify any misspecifications and correct them inorder to augment the research quality (Charles, 2020).

The multicollinearity problem can be measured by the VIF in the collection of several regression variables. The Breusch-Pagan/Cook Weisberg test is applied to detect the heteroscedasticity problem.

Serial-correlation tests should be apply to macro-panels with extended time series data (20–30 years), but not a problem in micro-panels (with a few years). The basic assumption of OL square is non-autocorrelation or absence of serial-correlation. This assumption tells us the error term at time t is not serially correlated with the error term at any other point time. Autocorrelation is detected by the Breusch-Godfrey LM Test.

A normal distribution is one that is not skewed and has a kurtosis coefficient of three. Jarque Bera formalizes this by checking for normality in the residuals and determining whether the coefficient of skewness and kurtosis are zero and three, respectively. Skewness is a characteristics or measures of how a distribution is normal or not symmetric about its mean value, while kurtosis is a characteristics of how fat the distribution’s tails are. Even at a 5% significant level, the Bera-Jarque probability statistics/P-value is not likely to be significant (Bonato and Nowakowski, 2011).

According to Baltagi et al. (2013), cross-sectional dependence (CD) is a difficult in macro panels for extended time series data, In micro panels, this isn’t a big deal (a large number of cases and a few years). The null hypothesis in the Pasaran CD tests is that residuals across entities are uncorrelated, while the alternative hypothesis in the Pasaran CD tests is that residuals across entities are correlated and CD may cause test results to be skewed also called contemporaneous correlation.

## 3 Results

### 3.1 Descriptive analysis

Profitability is measured by the ROA, which has a mean value of 15.14 percent per annum. This indicates that the sample industries' average incomes before taxes and interest were 15.14 percent. The maximum and the minimum value of ROA were 31.5 and 0.05 percent, respectively. That means the least and most profitable industries in manufacturing in Ethiopia earned 0.05% and 31.5% of the net earning of the assets of the firm, respectively. The employment growth measured by the an employee number which is a mean value of 15.26%. The smallest and largest value of this variable is −10%–31% (Table 1).

**Table 1:** Medium and Large size manufacturing industry of categories.

1	Manufacturer of food products	9	Manufacturer of motor vehicles
2	>> textiles	10	>> chemicals product
3	>> wood products excluding furniture	11	>> leather products
4	>> furniture	12	>> wearing apparel products
5	>> paper and printing	13	>> tobacco products
6	>> basic steel and iron	14	>> non-metallic product
7	>> machinery and equipment	15	>> Manufacturer of fabricated metal
8	>> basic metal

**Table 2:** Descriptive analysis of time varing and time invariant covariates by percent.

Variable	Mean	Median	Max	Min		Std.	Skewness	Kurtosis	Obs.
ADVI	9.5630	10.00	22.800	0.00		6.6617	0.0834	2.0389	150
EMG	15.2645	15.000	31.00	-10.40		8.820	0.0088	2.5273	150
F-size	16.830	16.000	39.300	0.1200		7.5716	0.3655	3.2595	150
IMP	27.500	25.700	52.40	5.500		9.8363	0.45065	2.7332	150
KPI	29.0313	30.000	60.000	10.000		8.2390	-0.06712	3.2837	150
ROA	15.1421	16.350	31.50	0.050	8.4386		0.34417	3.557	150

Advertising is measured by advertising intensity divided by total sales, it has a mean value of 9.56% with a minimum and maximum value of 0.0% and 22.8%, respectively, with the SD of the 6.66%. The minimum and maximum value of the firm size are 0.12 and 39.30%, respectively, with a mean value of 16.83%. The minimum and maximum value of the Imported are 5.5 and 52.4 present, respectively, with a mean value of 27.5% (Table 1).

**Figure 1:**
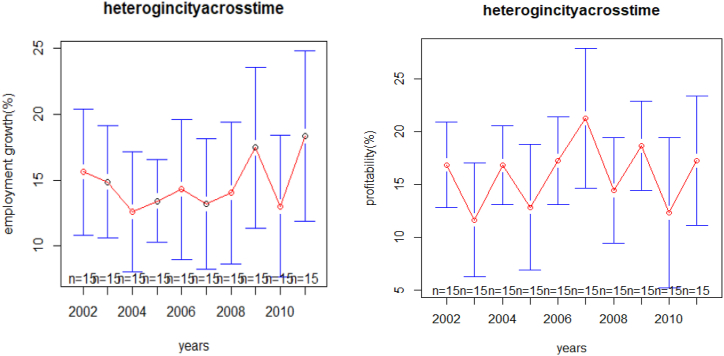
Heterogeneity of industries on employment growth and Profitability across the time.

As shown in Figure 1, mean plots of the profitability and employment growth of the medium and large-scale manufacturing industries from 2002 to 2011 E.C, the randomness pattern of employment growth from 2002 to 2004 shows a slight decrement sign, where as from 2009 to 2010 it shows a decrement sign. However, from 2007 to 2009 and 2010 to 2011, high employment growth with compared to the other years. In Figure 1, from 2010 to 2011, the randomness pattern for one year to another year showed depressed and upward in a slightly uniform pattern of profitability, but from 2005 to 2007, it shows a high increment percentage of profitability.

In Figure 1, the large error bars indicated that there were high range of employment growth and profitability among industries in the same year.

**Figure 2:**
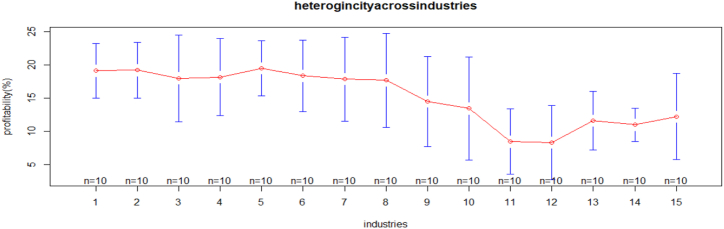
Heterogeneity across industries on profitability.

**Figure 3:**
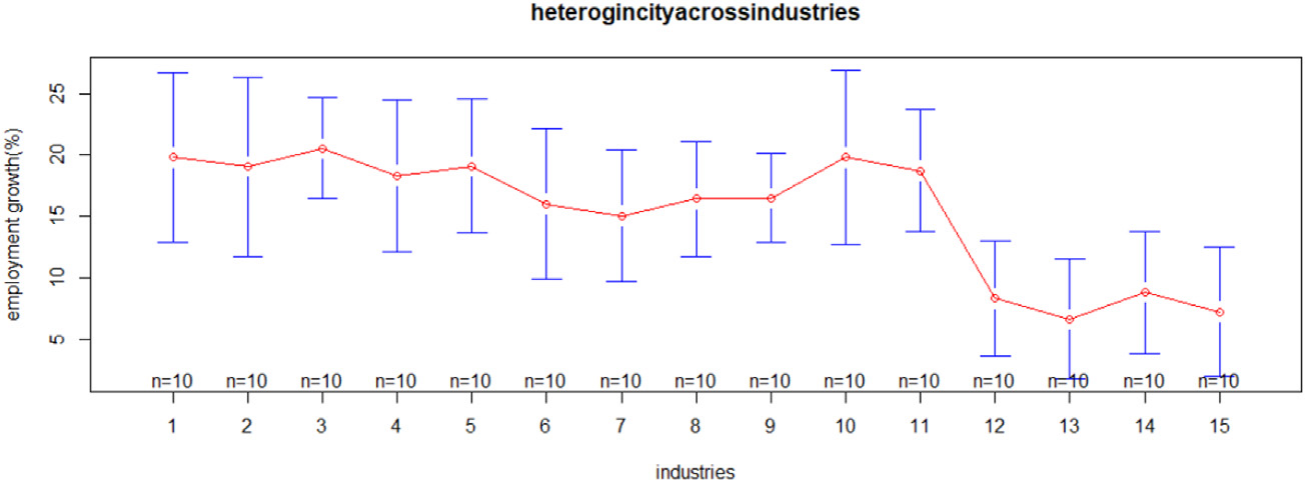
Heterogeneity across industries on employment.

Figure 2 indicates that the profitability of medium and large size manufacturing industries is heterogeneous. The number of manufacturing industries, whose names are mentioned in Table 2, from industries 1 to 8, the mean plots show a uniform pattern of industries' profitability. While from industries 8 to 13, the mean plots show a heterogenous pattern of industries' profitability. In addition, industry five, manufacturers of paper and printing, has good profitability compared to the other industries. Figure 3, from industries 1 to 10, shows a slightly uniform pattern, whereas from industries 10 to 13, it shows the heterogeneity of industries on employment growth. In addition, industry three, which manufactures wood products other than furniture, and industry ten, which manufactures chemical products, have experienced strong economic growth in comparison to the other industries.

### 3.2 Econometric analysis

The result of Table 3 shows that based on the Lagrange multiplier test, the random model is reasonable model compared to pooled OLS on profitability and employment growth.

**Table 3:** Lagrange multiplier test.

On Profitability		On employment growth	
F-statistics	p-value	F-statistics	p-value
3.3189	(p < 000)	2.4703	0.00675

As presented in Appendix 1, the value of Adj. R-square within the estimation method, FD, and LSD were 0.5532, 0.5952, and 0.5532 respectively on profitability. Thus, the FD estimation method was better than the LSD, and the within-estimation method eliminated the industrial effect on profitability since the value of Adj. R-square for FD was greater than the others, on employment growth, the within-group estimation method was better than the first difference and LSD estimation methods.

The chow test statistic for individual effect in Table 4 shows that the pooled OLS model is consistent for profitability. Table 4 presented data on employment growth, which means the fixed model is consistent. Therefore, based on the chow test, the fixed model was an appropriate model compared to pooled OLS model for employment growth, but pooled OLS model better compare to fixed model for profitability.

**Table 4:** Chow test for individual effects.

On Profitability		On employment growth	
F-statistics	p-value	F-statistics	p-value
F1 =-2.863 and F2 = 120	1	F1 = 2.272 and F2 = 120	0.0087

Table 5 results show that the p-value was 0.624. Then, the hull hypothesis were accepted, since there was a structural break point in the data, the random model was reasonable model than the fixed model on profitability. In the result of employment growth presented, the p-value is 0.42. As a result, profitability is interpreted similarly. As shown in Table 5, five industries influence on profitability and four on the industry’s influence employment growth. Thus, based on the theory of Yang and Land, the random model was a reasonable model to fit the data well than the fixed effect model.

**Table 5:** Hausman test.

On Profitability		On employment growth	
Chi-square	p-value	Chi-square	p-value
11.779	0.624	15.565	0.4115

Generally, the study was used for two response variables and also, by using three tests of model selection, which means, the LM-test, the chow test, and the Hausman test, showed that the random model was a reasonable model to fit the data compared to the pooled OLS and fixed effect on both profitability and employment growth.

Table 6 indicates the correlation matrix of variables. So the result showed there is no pairwise relationship among the independent variables because the correlation result of each independent variable is less than 0.25, except the variable import on advertising, and the correlation of profitability of employment growth was 0.21%, which means the two outcome variables were weeks apart. Therefore, the results point out that the problem of multicollinearity did not exist between independent attributes in the model. Hence, all the variables were retained and used in the estimations.

**Table 6:** Correlation matrix of the variables.

	Advertising	Employment	F-size	Import	Capital	Profitability
Advertising	1.0000					
Employment	0.551326	1.0000				
F-size	0.207810	0.666387	1.0000			
Import	0.257987	0.746886	0.223359	1.0000		
Capital	0.050665	0.418824	0.034251	0.096968	1.0000	
Profitability	0.72921	0.206374	0.67644	0.750513	0.371063	1.0000

Table 7, the result showed the VI factor of each of these explanatory variables were far less than 10, which implied no need to suspect multicollinearity in the model.

**Table 7:** Variance inflation factor.

Variable	Import	Advertising	F-size	Government	Ownership	Capital
**VIF**	1.19	1.38	1.09	1.04	1.09	1.04

As shown in Table 8, both the employment growth and profitability tests give the same conclusion: there is no evidence for the presence of heteroscedasticity since the p-values in both cases were above 0.05.

**Table 8:** Breusch-Pagan/cook Weisberg test.

On Profitability		On employment growth	
BP	p-value	BP	p-value
22.15	0.1039	24.34	0.05954

Table 9 shows that the Breush-Godfrey LM test gives an F-statistic of 24.91 with a probability of 0.06 on profitability and an F-statistic of 6.67 with a probability of 0.67 on employment growth. Hence, from both response variables of the test or estimate, we have evidence to accept no autocorrelation at a 5% significance level.

**Table 9:** Breusch-Godfrey correlation Lagrange Multiplier test.

On Profitability		On employment growth	
Chi-sq.	p-value	Chi-sq.	p-value
24.91	0.06116	6.668	0.7563

**Figure 4:**
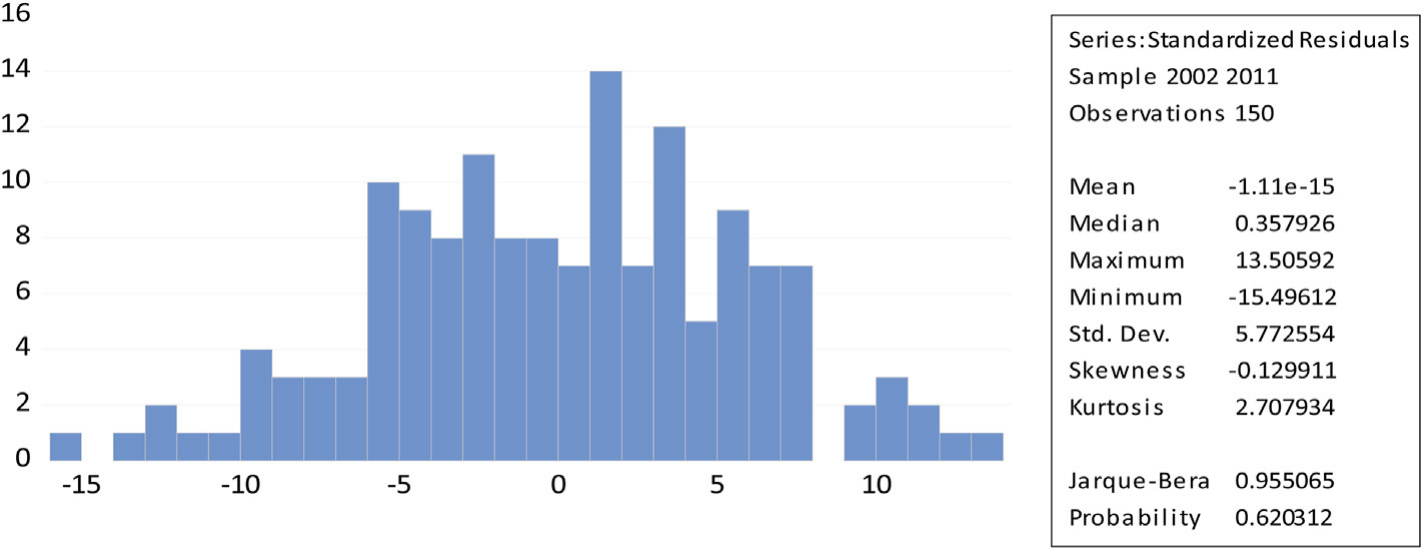
Normality of Profitability of normality.

**Figure 5:**
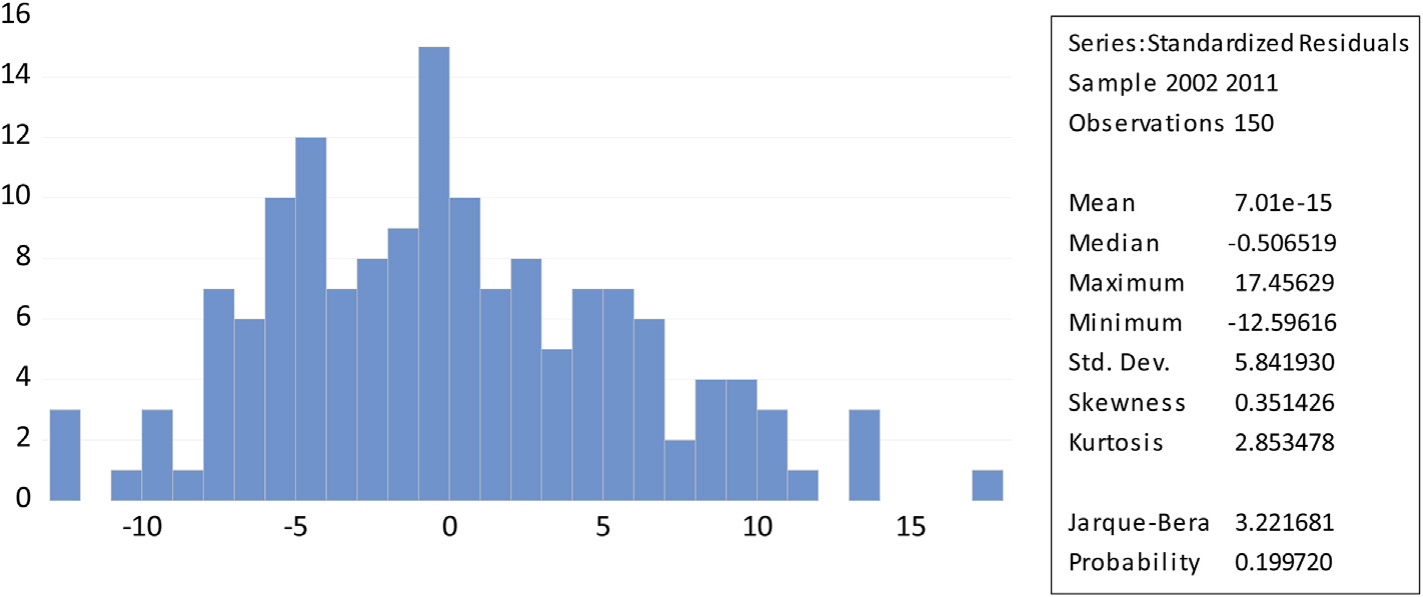
Normality of employment growth.

Figure 4 shows that the skewness was close to zero (−0.13) and the kurtosis was less than three (2.708), respectively, and the value of probability was 0.62, which is greater than the 0.05 level of significance. Hence, we have evidence that the profitability normality model assumption is reasonable. According to Figure 5, it shows that the p-value was 0.199. As a result, we have evidence that the employment growth normality model assumption is reasonable at the 5% level of significance.

According to Table 10, cross sectional dependency tests don’t reject under the null postulate for both employment growth and profitability since the p-values of 0.60 and 0.54 were greater than 5% level of significant, respectively. Hence, we have enough confirmation suggesting the absence of cross-section independence in the model and observations across cross-sectional units are independent, which gives the estimator of random a consistent and efficient method.

**Table 10:** Pesaran’s test of cross sectional dependency.

On Profitability		On employment growth	
Pesaran’s test	p-value	Pesaran’s test	p-value
0.524	0.6005	-0.616	0.5380

R-square is not very informative in panel data analysis. Because in the panel data, there is heterogeneity of individuals cross sections, R-square is low in cross-sectional data to time series. If your panel data is long run or more time dominant, R-square will be higher as related to the case while panel data is more cross section dominant or short run data (Soylu et al., 2018). Table 11 shows the model summary for the random model.

**Table 11:** The value of overall R-square.

On employment		On Profitability
R-Square		R-Square
Within	0.4925	0.6357
Between	0.7617	0.5848
Overall	0.5537	0.6215

Table 12 shows that the unit-specific error term ($\sigma_{u}$) is 1.612, whereas the SD of residuals or overall error term ($\sigma_{\varepsilon }$) is 5.01; and we assume that the association between the specific error component (u) and regressors (x) is zero by assertion. In addition, the value of the proportion of variance because of unobserved specific effect (rho) is 0.094, which means 9.4% of the variance is because of the differences across panels. The random model estimated coefficients of firm size, ownership, import intensity and advertising intensity were significant and positive impact on profitability, while government has significant effects with negative influence. .

**Table 12:** Profitability for Random-effects FGLS regression.

IDV	Estimate	Std. Err.	z-value	P>|z|	[95% Conf. Interval]
Intercept	4.437051	2.868504	1.55	0.122	-1.185112, 10.05922
Capital	0.0645369	0.057476	0.12	0.906	-.1032105, .1164754
Import	.1024126	.0502196	2.04	0.041	.003984, .2008411
Firm size	.1399453	.066465	2.11	0.035	.0096763, .2702143
Government (gov.t)	-6.749317	.9220354	-7.32	0.000	8.556474, –4.942161
Ownership (private)	8.940028	1.179853	7.58	0.000	6.627558, 11.2525
Advertising	.2777572	.0860232	3.23	0.001	.1091549, .4463594
year 2003	-2.911184	1.7884	1.43	0.141	- 0.947026, 6.063371
year 2004	0.6694374	2.019645	-0.50	0.619	-4.961646, 2.955217
year 2005	-1.857452	1.687974	1.20	0.231	-1.286375, 5.330362
year 2006	1.174455	2.36052	0.95	0.341	-2.377762, 6.752712
year 2007	1.447533	1.673289	2.08	0.488	-2.639291, 5.534357
year 2008	-2.23223	2.233865	1.18	0.240	-1.751821, 7.004767
year 2009	-.7425887	2.062194	0.96	0.338	-2.067698, 6.015953
year 2010	-4.595528	1.985811	-2.31	0.213	-8.487645, 0.703411
year 2011	-3.138808	2.199642	-1.56	0.119	-4.16285, 9.207201
sigma_u	1.7030664				
sigma_ε	5.3191699				
$corr\left(u_{i},X\right)$	= 0 (assumed)				
Rho	0.09298053 (fraction of variance (u_i))				

Table 13 indicates that the estimate of the unit-specific error term ($\sigma_{u}$) is 1.26 and the overall error term ($\sigma_{\varepsilon }$) is 5.84. The assertion assumes that the association between u and x is zero. In addition, the value of “rho” is 0.045, which means 4.5% of the variance is as consequences of the differences across panels. All the independent variables have a significant effects, except capital intensity and ownership, with positive influence except import intensity. The left ones have a significant influence on the employment growth of Ethiopian manufacturing industries. All dummy of ten years have insignificant effect on employment growth (Table 13).

**Table 13:** Employment for Random-effects FGLS regression.

IDV	Estimate	Std. Error	t-value	P>|t|	[95% Conf. Interval]
Intercept	4.544199	3.283575	1.38	0.166	-1.891489, 10.97989
Capital	.0865772	.0646764	1.34	0.181	-.0401861, .2133405
Import	-.1271445	.0575153	-2.21	0.027	-.2398725, –.0144165
Firm size	.259479	.0767951	3.38	0.001	.1089633, .4099947
Government (gov.t)	9.225367	1.067393	8.64	0.000	7.133315, 11.31742
Ownership (private)	-.9974812	1.359465	-0.73	0.463	-3.661983, 1.667021
Advertising	.3675213	.0997868	3.68	0.001	.1719428, .5630997
Year 2003	2.022054	2.541374	0.96	0.258	-7.854536, 2.107466
Year 2004	-0.625148	2.085655	-0.30	0.411	-4.807373, 1.96412
year 2005	-.3003043	2.399582	-0.14	0.180	-7.922952, 1.483235
year 2006	-1.052462	2.135576	-0.49	0.378	-6.92667, 2.628825
year 2007	-4.002579	2.117371	-1.89	0.800	-4.840594, 3.731173
year 2008	1.384798	2.16814	-2.17	0.122	7.651705, .9037763
year 2009	1.341173	2.093893	0.64	0.185	-8.758541, 1.689674
year 2010	-0.0944029	2.09802	-0.04	0.328	-7.652052, 2.556051
year 2011	2.699156	2.129114	1.27	0.425	-6.250868, 2.635284
sigma_u	1.2632672				
sigma_ε	5.8354371				
$corr\left(u_{i},X\right)$	= 0 (assumed)				
rho	.04476651 (fraction of variance (u_i)				

Table 14 shows the effect of industries on profitability and employment growth. Then, the results exhibited that Wearing apparel, leather, non-metallic, fabricated metal, and tobacco have significant effects and have a negative influence on profitability. On employment growth, the results revealed that Wearing apparel, fabricated metal, non-metallic ​and tobacco have significant effects and a negative influence (Table 14).

**Table 14:** Industries on employment and profitability growth.

Industries	On profitability			On employment growth		
	Coef.	z	P>|z	Coef.	Z	P>|z
Textiles	.096	0.03	0.978	-.7479	-0.22	0.829
Wood products	-1.11	-0.32	0.752	.720	0.21	0.836
Furniture	-.9399	-0.27	0.789	-2.698	-0.78	0.437
Paper and printing	.370	0.11	0.916	-.720	-0.21	0.836
Iron and steel	-.7559	-0.21	0.83	-3.78	-1.09	0.276
Equipment	-1.247	-0.35	0.723	-4.745	-1.37	0.172
Basic metal	-1.43	-0.41	0.684	-3.39	-0.98	0.329
Motor vehicles	-4.61	-1.31	0.190	-3.33	-0.96	0.337
Chemicals product	-5.647	-1.60	0.109	-.0054	-0.00	0.999
Leather products	-10.636	-3.02	0.003	-1.08	-0.31	0.756
Wearing apparel	-10.804	-3.07	0.002	-11.50	-3.31	0.001
Tobacco	-7.5	-2.13	0.033	-13.17	-3.80	0.000
Non-metallic	-8.11	-2.30	0.021	-10.99	-3.17	0.002
Fabricated metal	-6.8946	-1.96	0.0490	-12.58	-3.62	0.000

## 4 Discussion

According to Table 12, the random model, import intensity had a significant effects with positive influence on profitability over the period 2002-2011E.C. The results was concur with Haftu (2019), inconsistent with other similar studies (Dai and Wang, 2014; Alem et al., 2016). Firm size used had significant contribution on profitability in Ethiopia (p-value <0.001). The positive impact of industry or size use on profitability is consistent with other similar studies in Ethiopia (Dagim, 2020; Haftu, 2019), but inconsistent with other studies (Kidanu, 2016; Woldemariam, 2017). Furthermore, the study of (Alafita-Vásquez et al., 2021) discovered that firm size can have a significant effect and positive impact by using fixed effect, pooled OLS, random effect, and system-GMM models; however, based on the model selection criteria, the Hausman test, Hausman (1978), compares the random versus fixed effects, under the null hypothesis, the individual influence are independent with the explanatory variables in the model (Amini et al., 2012). The LM Test determine whether a random effect or a simple OLS regression should be implemented. The null hypothesis claim there is no visible difference across cross-sectional units, only the random effect estimate is consistent with this study, the ownership structure had a significant impact and a positive effect on profitability (Table 12).

According to microeconomic theories, before continuing their expansion with less profitable ones. As a company increases in size, it suits more difficult for its managers to rise its profitability (Steffens et al., 2009). As a firm increase in size, more organizational and management complexity emerges, diverting managers' attention away from controlling operational expenses and resulting in a decline in profit rate. As a result, profitability cannot be achieved through just growth. Evolutionary theories on business growth, led by the study by (Cornell and Kalt, 2005).

Based the random effect estimated result, in Table 13, the import intensity coefficient, estimated at −.127 was highly significant and had a negative influence on employment, p-value < 0.027, the random model results reveals that industry size and advertising have greatly contributed to employment growth (p-value < 0.001). Based on the random model results, the use of advertising and government had a significant positive impact on Ethiopian employment growth (Table 13).

Table 12, The significant influence of use of advertising on profitability was consistent with other studies (Srinivasan and Hanssens, 2009; Graham and Frankenberger, 2000; Taylor, 2015; Dagim, 2020). In the study (Alafita-Vásquez et al., 2021), there was consistent confirmation of the correlation between advertising intensity and profitability on the use of total firm of large and medium-scale manufacturing companies. Capital intensity had no significant for random effect on profitability. The capital intensity is positive influence on profitability was consistent with other studies (Kidanu, 2016) but inconsistent with other studies (Degefa, 2017), (Woldemariam, 2017) and (Raheman et al., 2010). The study by (Alafita-Vásquez et al., 2021) using static panel models (pooled OLS, RE, and FE) found the relationship among the capital profitability and structure of LMSMI in Ethiopia was inconsistent. Ownership used had significant random effect contribution on profitability in Ethiopia (p-value < 0.001). The positive influence of ownership and significant effect use on profitability is consistent with other similar studies such (Arouri et al., 2014) and (Degefa, 2017) but inconsistent with other studies (Roe, 2002) and (Villalonga, 2019) (Table 13). Actually, we did find a significant influence between the type of ownership and profitability was consistent with (Alafita-Vásquez et al., 2021) but inconsistent with the coefficient on profitability.

The relationship between both growth and profit is at the core of major theories like the theory of financial and economic development (Fritsch, 2017), the theory of entrepreneurship (private enterprise) and firm growth (Penrose and Penrose, 2009). Currently, many economists accept maximizing profit and economic growth are two competitor objectives within an industry (Jang and Park, 2011). Several theories support the positive relationship across profitability and employment growth. Business growth is measured to guide towards a decrease in network externalities, outsourcing, costs through economies of scale, and a growth in mediation power with providers and clients (Markman and Gartner, 2002).

According to Table 13 results, import intensity had negative and significant impact on employment growth in the random model. The negative influence of import amount on employment growth was supported by other studies (Asare et al., 2017), but inconsistent with other studies (Yayeh, 2018). According to Table 13 results, firm size had a significant random model on employment growth, and its positive influence on growth was consistent with other similar studies (Bigsten and Söderbom, 2011). The positive influence of capital intensity and insignificant effect use on employment growth is consistent with other similar studies such (Yayeh, 2018) but inconsistent with other studies (Singh and Asress, 2011) (Table 13).

Appendix 1 shows that the correlation between employment and profitability was positive and had an insignificant influence on growth, which is consistent with other studies (Federico and Capelleras, 2015), and (Geroski and Mazzucato, 2002). However, this finding contradicts previous research (Pachauri et al., 2014; Lee, 2018; Coad et al., 2013).

The panel model results indicate a significant and affirmative correlation between import intensity and manufacturing firm profitability at the 5% level of significance. This finding was coinciding with (Tavassoli and Jienwatcharamongkhol, 2014). As a result, the alternative hypothesis that import intensity has a positive influence and significant impact on the growth of Ethiopian manufacturing industry is supported.

## 5 Conclusions

The study considered to determines the profitability and employment growth of medium and large size manufacturing industries from the period 2002 to 2011. Findings of this study indicated that employment growth and profitability are influenced by import intensity, firm-size, capital intensity, advertising, ownership structure, and government. This chapter summaries the conclusions and recommendations, as well as suggestions for future studies.

Manufacturing industries are great contributors to the growth and development of any country in the world. Their role has become vital in developing countries like Ethiopia, where there have numerous support services are provided by sectors and government agencies aimed at fostering the development of manufacturing industries.

The study used a random model for six variables; capital intensity, firm size, import intensity, advertising, ownership structure, and government. The results revealed that the used Jarque-Bera test verified that residuals are approximately normally distributed, while the Breusch-pagan or cook weisberg test and Breusch-Godfrey LM test confirmed that residuals do not exhibit heteroscedasticity and autocorrelation respectively.

Based on the random model result, ownership, firm-size, advertising and import intensity have a statistically significant impact and positive relationship on profitability, while government has a significant effects but negative impact. In addition to that Wearing apparel, leather, non-metallic, fabricated metal and tobacco have a significant effect and negative influence on profitability. Generally, any decrease or increase in import intensity, firm-size, ownership structure, government, advertising intensity to decrease or increase in the profitability of the sampled industries.

Based on the random model result, firm-size, government, and advertising intensity have statistically significant impacts and positive relationships on employment growth; while capital intensity and ownership have statistically insignificant effects. However, ownership and import intensity have a negative influence. Therefore, the import intensity, firm-size, government, advertising on the employment growth of Ethiopian manufacturing industries have a statistically significant impact at 95% confidence level.

Finally, the overall R-square value coefficient of employment growth and profitability are 62.15% and 55.37%, respectively, which means the dependent variable of employment growth or profitability of Ethiopian manufacturing industries was well explained by the six independent variables that are considered in the model.

Based on the research findings, the following reachable recommendations were forwarded:

• Private ownership has a significant influence on the profitability of the Ethiopian LMSM sector. Thus, all the responsible and concerned bodies should support private firms or industries to improve profitability.
• From a policy perspective, the larger industries should have been targeted in order to increase jobs. The implication is that large firms are more important for development than small firms since they can generate more employment. Hence, policymakers and or the government manufacturing sector should strengthen their performance or efficiency that improve advertising intensity.
• Based on the study results and inference of the study, the authors strongly recommends that the manufacturing industry sector and the Ethiopian government should be reduce import intensity to improve profitability and employment growth.
• The LMSMI sector of Ethiopia should give attention to the industries of machinery, leather, non-metallic, fabricated metal, and tobacco.

The basic key point that is necessary, but could not be included in this research and will be done by the next researcher is:

• In the future, researchers will need to work on adding period and other variables to better identify the factors that are influencing the profitability and employment growth of the relationship.

## 6 Limitation of the study

The study examined only the stated employment growth and profitability across industries, while there are other variables such as management skill which also have an influence on firm performance (Jacobson et al., 2009). Shortage of comprehensive data on certain attributes such as, research and development and skills of workers, which are expected to be one of the most decisive, determine the industry’s profitability and employment growth. There is a lack of empirical work or literature on some independent variables, includes industry asset depreciation rate and government rule and regulation for both profitability and employment growth.

## Declarations

### Author contribution statement

Aragaw Eshetie Aguade, Dasash Ayanaw, Endeshaw Assefa Derso:Conceived and designed the experiments; the acquisition of data, or the analysis and interpretation of data; Performed the experiments, Contributed reagents, materials, analysis tools or data; Wrote the paper.

### Funding statement

This research did not receive any specific grant from funding agencies in the public, commercial, or not-for-profit sectors.

### Data availability statement

Data will be made available on request.

### Declaration of interests statement

The authors declare no conflict of interest.

### Additional information

No additional information is available for this paper.

## Appendix A Supplementary data

Supplimentary Supplimentary
