# Supplementary material for: Panel data analysis of profitability and employment growth of medium and large size industries in Ethiopia
Source: Heliyon. 2022 Oct 4;8(10):e10859. doi: 10.1016/j.heliyon.2022.e10859 (PMC9562453; doi:10.1016/j.heliyon.2022.e10859)
Supplement: Supplimentary [file mmc1.pdf]

APPENDIX -I

1. Summary of fixed effect model

On Profitability			On employment	
**Estimation method**	R-Squared	Adj. R-Squared	R-Squared	Adj.R-Squared
**FD**	0.6375	0.5952	0.49775	0.37637
**Within**	0.6402	0.5532	0.5010	0.44279
**LSD**	0.6402	0.5532	0.49775	0.375

Estimation methods of fixed effect model on profitability and employment growth

First different for on profitability

Independent variables	Estimate	Std. Error	t-value	P>|t|	[95% Conf. Interval]
Intercept	-0.25976	0.636306	-0.408	0.683	-1.506902 0.987374
Capital	0.00032	0.00065	0.499	0.618	-0.0009547 0.00160
Import	0.38914	0.05626	6.916	0.000	0.2788692 0.49941
F-size	0.15965	0.05568	2.867	0.004	0.0505162 0.26879
Factor(GOV)non	-4.13536	1.37070	-3.017	0.002	-6.8218950 -1.44883
Factor(OWN)public	-4.36621	1.405475	-3.106	0.002	-7.1208933 -1.61153
Advertising	0.05297	0.054866	0.965	0.335	-0.0545572 0.16051

First different for on Employment growth

The independent variables:	Dependent variable is employment growth				
	Estimate	Std. Error	t-value	P>|t|	[95% Conf. Interval]
Intercept	-0.091859	0.879303	-0.104	0.916	-1.815262 1.631543
Capital	0.000487	0.000931	0.5242	0.173	-0.001336 0.002312
Import	0.186744	0.074577	0.7664	0.000	0.040575 0.332913
F-size	0.053433	0.053433	-2.999	0.000	0.0930455 0.325926
Factor (GOV) non	5.942750	1.981608	-1.999	0.284	-9.826632 2.05886
Factor(OWN) public	2.804057	2.784194	1.007	0.001	2.021054 7.83849
Advertising	0.636568	0.080288	7.9285	0.000	0.4792065 0.7939309

Within-group estimation on profitability

Independent variables	The dependent variable is profitability				
	Estimate	Std. Error	t-value	P>|t|	[95% Conf. Interval]
Capital	5.18e-05	5.73e-04	0.090	0.928	-0.002292,4.2475e-05

Import	4.17e-01	5.62e-02	3.633	0.000	0.264455, 4.6858e-01
F-size	1.90e-01	5.25e-02	5.820	0.000	0.190742,3.8445e-01
Factor(GOV)non	-3.5700	1.1110	-3.214	0.001	-4.02167, 6.7559e-01
Factor(OWN)public	-3.5700	1.1513	-3.106	0.002	-5.51884,-5.5733e-01
Advertising	1.04e-01	4.95e-02	2.114	0.035	0.031709, 2.6202e-01

Within group on employment growth

Independent variables	Dependent variable is employment<br>Estimate	Std. Error	t-value	P>|t|	[95% Conf. Interval]
Capital	0.000998	0.000867	1.1508	0.251	-0.000702, 0.0026990
Import	0.326831	0.075862	4.308	0.001	0.178143, 0.475520
Firm size	0.108130	0.071986	1.502	0.000	0.032960, 0.249221
Factor(GOV)non	6.020379	1.745697	-3.448	0.3240	-9.4418834, 2.598875
Factor(OWN)public	2.853944	1.843902	1.547	0.000	0.7600392, 6.467927
Advertising	0.441547	0.085611	5.157	0.000	0.2737516, 0.609342

Profitability for LSD estimation method

IDV	Estimate	Std. Error	t-value	P>|t|	[95% Conf. Interval]
Capital	0.00041	0.0005691	0.07	0.943	-0.0010749, 0 .00116
Import	-0.2515941	0.0639824	3.93	0.016	0.220012, 0.2156013
Firm size	0.430967	.0627401	6.87	0.000	0.3071181, 5291906
Government(gov.t)	6.610327	1.108120	6.31	0.000	0.0930467, 0.298707
Ownership(private)	1.066492	1.099920	0.99	0.003	-5.63068, -1.126045
Advertising	0.1957263	.057239	3.42	0.002	-5.573378, -1.228494
Industries_2	0.369	2.541374	0.96	0.258	-8.852, 2.173458
Industries_3	-10.525	2.085655	-0.30	0.411	-4.825788, 5.942558
Industries_4	-6.761	2.399582	-0.14	0.180	-3.752598, 7.269293
Industries_5	0.079	2.135576	-0.49	0.378	-7.501363, 3.447842
Industries_6	0.102	2.117371	-1.89	0.800	-5.34464, 6.08279
Industries_7	-0.654	2.16814	-2.17	0.122	-4.918548, 5.636051
Industries_8	-3.261	2.093893	0.64	0.185	-4.083518, 6.893431
Industries_9	-4.298	2.09802	-0.04	0.328	-5.071269, 6.373945
Industries_10	-9.287	2.129114	1.27	0.425	-6.250868, 2.635284
Industries_11	0.743	2.942708	-1.97	0.049	-11.55051, -0.1530568

Industries_12	0.521	2.80829	-1.63	0.103	-10.07692, 0.9313759
Industries_13	-9.231	2.752501	1.20	0.231	-2.100605, 8.682584
Industries_14	-7.620	2.9721	0.96	0.336	-2.967834, 8.682584
Industries_15	1.041	2.89354	-0.62	0.537	-7.458262, 3.884223

Employment growth for LSD estimation method

IDV	Estimate	Std. Error	t-value	P>|t|	[95% Conf. Interval]
Capital	0.005725	.0005725	0.78	0.097	-.009113, 0 .230563
Import	-0.2015941	0.0639824	2.93	0.000	0.128849, 0.3647221
Firm size	0.330967	.0627401	6.87	0.003	0.312158, 0.529153
Government(gov.t)	5.610327	1.108120	1.31	0.000	2.738478 6.893213
Ownership(private)	2.066492	1.099920	3.99	0.000	1.047801 3.180785
Advertising	0.1057263	.057239	8.42	0.225	0.0835398, 0.3079127
Industries_2	2.8700	0.788	0.96	0.258	-7.854536, 2.107466
Industries_3	-9.005	-2.473	-0.30	0.411	-4.807373, 1.96412
Industries_4	-8.022	-2.203	-0.14	0.180	-7.922952, 1.483235
Industries_5	1.3500	0.371	-0.49	0.378	-6.92667, 2.628825
Industries_6	-1.340	-0.368	-1.89	0.800	-4.840594 3.731173
Industries_7	1.3400	0.368	-2.17	0.012	7.651705 .9037763
Industries_8	0.9440	0.259	0.64	0.185	-8.758541, 1.689674
Industries_9	0.3845	0.259	-0.04	0.328	-7.652052, 2.556051
Industries_10	-4.940	-1.356	1.27	0.425	-6.250868, 2.635284
Industries_11	0.5200	0.143	1.66	0.097	-.019113, 0 .230563
Industries_12	3.0420	0.835	3.93	0.000	0.128849, 0.3647221
Industries_13	-9.860	-2.707	6.87	0.000	0.312158, 0.529153
Industries_14	-10.10	-2.776	6.31	0.000	2.738478 6.893213
Industries_15	3.4200	0.939	0.99	0.323	-1.047801 3.180785

1. **Employment on Profitability**

	**Coef.**	**Std.**	**Z-value**	**Sig.**
**Intercept**	15.300834	1.60487	9.5341	<2e-16 ***
**profitability**	0.04046	0.07823	0.5172	0.605
